# Exploring the application of generative artificial intelligence in nursing: a cross-sectional study

**DOI:** 10.3389/fpubh.2026.1689418

**Published:** 2026-01-28

**Authors:** Jinling Wu, Mengmeng Yang, Xiujuan Wei, Yanan Zheng, Jiexin Deng

**Affiliations:** 1 School of Nursing and Health, Henan University, Kaifeng, China; 2 First Medical Center of the PLA General Hospital, Beijing, China; 3 Beixiaguan Community Health Service Center, Beijing, China; 4 Southern Medical Branch of the PLA General Hospital, Beijing, China

**Keywords:** generative artificial intelligence, nursing field, current state of application, nursing staff, cross-sectional survey

## Abstract

**Objectives:**

This study aims to systematically investigate the application of Generative Artificial Intelligence (GAI) in nursing practice within China. It seeks to map current usage patterns, identify perceived benefits and implementation challenges, and uncover the functional needs of nursing staff regarding GAI. Additionally, the research will assess the real-world performance and adoption of emerging local GAI platforms. The findings are expected to provide foundational evidence to guide the scientifically sound and contextually appropriate development of GAI in nursing.

**Methods:**

The convenience sampling method was used to select nurse interns, staff nurses, and nurse managers from 20 provinces between April and June 2025 as survey respondents. We designed the questionnaire through a literature review as well as evidence extraction. A panel of experts assessed the content validity of the questionnaire.

**Results:**

According to a survey of 181 nurses, GAI has achieved a high adoption rate (92.81%), which was not significantly associated with any demographic factors (all *p* > 0.05). Further analysis, however, revealed that seniority was a significant predictor of usage frequency (*B* = 0.507, *p* = 0.001). In terms of platform preference, locally developed GAI platforms, such as DeepSeek and Doubao, dominated the landscape, collectively accounting for over 60% of total usage. The most prevalent application was the generation of health education materials (61.30%). A key finding to emerge from the data is the role-specific utility of GAI: it reduced administrative workload for nurse managers, enhanced workflow efficiency for clinical nurses, and fostered innovation among nursing interns. Despite this promising adoption, significant challenges remain, primarily concerns regarding the professional accuracy of AI-generated content (40.96%) and its potential impact on clinical autonomy.

**Conclusion:**

This study establishes that GAI adoption among Chinese nurses is widespread but nuanced, marked by a preference for local platforms and role-specific benefits. The primary barriers to integration—concerns about content accuracy and clinical autonomy—underscore the need for clinically validated and trustworthy systems. These findings advocate for targeted training and ethically aware design to fully realize GAI’s potential as a collaborative tool in nursing.

## 1 Introduction

Generative artificial intelligence (GAI) is revolutionizing digital content creation by producing high-quality, human-like outputs (1). In healthcare, the rapid advancement of AI technology is fostering innovations in medical research and driving shifts in clinical practice (2). While studies indicate that GAI has the potential to improve efficiency in domains such as clinical documentation and diagnostic support, it also presents challenges, including model hallucinations, data biases, and regulatory ambiguities (3).

Concurrently, AI is reshaping education systems by broadening educational access and enabling personalized learning; however, it also introduces challenges pertaining to curriculum adaptation, assessment methods, data privacy, and algorithmic bias (4). In the nursing sector, AI applications are anticipated to mitigate time-consuming, routine tasks, thereby optimizing service delivery and reallocating nursing time toward direct patient care (5). Additional prospective benefits encompass process optimization (6), the delivery of personalized care (7), and enhanced healthcare accessibility (8). Corroborating this, a systematic review underscores AI’s potential to improve workflow efficiency and resource utilization within clinical nursing (9).

However, the practical integration of GAI faces several significant barriers, including insufficient region-specific data, constrained clinical performance, high operational costs, a lack of clear regulatory guidelines, and uneven resource distribution (10, 11). As emphasized by Reddy et al., successful deployment requires robust data protection protocols, human-in-the-loop oversight, and rigorous local validation (11). Although studies on nurses’ perceptions and attitudes toward AI have been conducted in several countries (12, 13), systematic investigations into the current application status and post-implementation effectiveness of GAI in nursing practice remain scarce. Furthermore, research specifically addressing the functional needs of nursing staff and offering development recommendations for GAI in this field is particularly limited (13).

Furthermore, research on the integration of GAI into the daily workflows of nurses in non-Western settings—particularly within China’s unique healthcare system—remains limited. Although local GAI platforms, such as DeepSeek and Doubao, are increasingly adopted owing to their open-source architecture and user-friendly interfaces, empirical evidence regarding their practical efficacy and depth of integration into nursing workflows is still lacking.

This study aims to address these research gaps through a systematic investigation of the GAI application landscape among nurses in China. Specifically, it examines context-specific usage patterns, assesses the real-world performance of local GAI platforms, and explores how nurses’ professional roles are evolving as they interact with the functional capabilities of these technologies. In doing so, the research moves beyond the prior literature’s primary focus on preliminary attitudes and adoption willingness.

## 2 Objects and methods

### 2.1 Subjects of the survey

From April to June 2025, nursing personnel from 20 provinces (cities) spread throughout six regions of China—East China, Central China, South China, Southwest China, North China, and Northeast China—were recruited as survey participants using convenience sampling.

Participants were included if they (a) participated voluntarily and (b) held one of the following professional roles: nursing managers with at least one year of experience, licensed staff nurses, or nursing interns who had completed a minimum of 3 months of clinical rotation. Exclusion criteria were (a) withdrawal from the study, (b) provision of incomplete survey responses, and (c) age under 18 years. The study protocol received approval from the Biomedical Research Ethics Subcommittee of Henan University (Approval No. HUSOM2025-703), and Informed consent was obtained from all participants.

### 2.2 Survey instruments

#### 2.2.1 Questionnaire development

The survey questionnaire was developed based on a systematic extraction of evidence from key literature. We first conducted a systematic review of major evidence-based clinical resources—including UpToDate, BMJ Best Practice, the Joanna Briggs Institute (JBI), the National Guideline Clearinghouse (NGC), and the Cochrane Library—as well as comprehensive bibliographic databases such as PubMed, Embase, Web of Science, the China National Knowledge Infrastructure (CNKI), and the Wanfang Database. Search strategies incorporated both controlled vocabulary (e.g., MeSH terms) and free-text keywords related to “Generative Artificial Intelligence,” “Chatbot,” “Large Language Model,” “Nursing,” and “Application.”

The literature search identified six key publications (14–19) that formed the basis for the questionnaire design. The final set of selected literature included three systematic reviews, two narrative reviews, and one clinical guidance document. All selected publications underwent a rigorous quality assessment and satisfied the predefined inclusion criteria.

Based on the synthesis of this literature, a structured framework was developed, encompassing six application indicators, 11 application limitations, and six potential contributions of GAI to nursing (Table 1). This framework subsequently informed the initial draft of the questionnaire.

**Table 1:** Literature summarizes the use of artificial intelligence in nursing.

Literature	Indicators of GAI application in nursing	Contributions of GAI to nursing	Limitations of the GAI application in nursing
Von Gerich (14)	Clinical decision-making	Improve information retrieval efficiency Improve work efficiency	Ethical challenges Insufficient training in GAI skills Risk of nurses being replaced Unclear definition of medical malpractice liability
O’Connor (15)	Clinical Decision-making Nursing Education	Improve work efficiency Assist clinical decision-making	Risks to the accuracy of information Ethical challenges Lack of trust in GAI among patients/families Insufficient training in GAI skills Unclear definition of medical malpractice liability
Seibert (16)	Nursing Education Patient self-management	Improve work efficiency Assist clinical decision-making	Risks to the accuracy of information Ethical challenges Lagging privacy protection Unclear definition of medical malpractice liability
King (17)	Clinical Decision-making Nursing Plan	Improve work efficiency Optimize patient communication Assist clinical decision-making	Risks to the accuracy of information Lagging privacy protection Ethical challenges
Wang (18)	Clinical decision-making Nursing education Scientific research Nursing plans	Improve work efficiency Enhance innovative thinking Assist clinical decision-making Reduce work stress Optimize patient communication	Ethical challenges Risks to the accuracy of information Lagging privacy protection Impact on independent judgment Lack of empathy in emotional support Unclear definition of medical malpractice liability
Kurniawan (19)	Patient self-management Health education	Improve work efficiency Assist clinical decision-making Optimize patient communication	Difficulty adapting to complex environments Risks to the accuracy of information Lagging privacy protection Lack of trust in GAI among patients/families

#### 2.2.2 Questionnaire refinement and structure

Based on the initial draft, a pilot test was administered to a convenience sample of 30 nursing professionals in Beijing, encompassing nurse interns, staff nurses, and nurse managers with diverse levels of clinical experience. Feedback regarding item ambiguity and clarity was incorporated to refine the wording, yielding the final version of the questionnaire.

The finalized instrument consisted of five sections: 1. Demographic Information (10 items on institutional and individual characteristics); 2. Current Status of GAI Application (10 items on GAI utilization at the individual level); 3. Departmental Integration of GAI Technology (3 items on current departmental adoption); 4. Evaluation and Perspectives on GAI (6 items on GAI evaluation, implementation barriers, and training interests); and 5. Open-Ended Questions (4 items on desired functionalities, perceived benefits, potential limitations, and general suggestions for GAI applications).

### 2.3 Validity assessment

To ensure the methodological rigor of the newly developed survey instrument, a formal content validity assessment was conducted. An expert panel comprising six nursing professionals with specialized backgrounds in smart nursing (*n* = 2), nursing management (*n* = 1), inpatient nursing (*n* = 2), and outpatient nursing (*n* = 1) was convened. Panel members had professional experience ranging from 18 to 26 years, held bachelor’s (*n* = 2) or master’s (*n* = 4) degrees, and included four senior-level and two intermediate-level professionals.

The assessment employed a standard 4-point rating scale (1 = not relevant, 4 = highly relevant) to evaluate each item’s relevance to the research topic of “generative artificial intelligence applications in nursing.” Results were quantified using both the Item-level Content Validity Index (I-CVI) and the Questionnaire-level Content Validity Index (S-CVI).

The analysis established robust content validity. I-CVI scores ranged from 0.833 to 1.000, exceeding the accepted threshold of 0.78 for item-level validity (20). The Questionnaire-level Content Validity Index/Universal Agreement (S-CVI/UA) reached 0.833, while the Questionnaire-level Content Validity Index/Average (S-CVI/Ave) was 0.972. These indices meet or surpass established benchmarks for satisfactory content validity (S-CVI/UA ≥ 0.8; S-CVI/Ave ≥ 0.90) (21, 22), confirming that the questionnaire demonstrates strong content validity at both item and overall instrument levels.

### 2.4 Data collection

The sample size was determined according to the Kendall (23)method, which recommends 5 to 10 times the number of questionnaire items. With 24 core items (excluding demographic questions) and an anticipated 20% rate of invalid responses, the minimum required sample size was calculated as 150. The study ultimately recruited 181 participants, exceeding this minimum threshold.

The questionnaire was administered electronically in a structured online format. Prior to commencing the survey, all participants were presented with an informed consent form. Only those who provided their digital consent were granted access to and could proceed with the subsequent questions. To improve response efficiency and maintain data integrity, technical restrictions were implemented to permit only one response per device. The questionnaire was configured to require responses for all closed-ended items and employed conditional branching, whereby subsequent questions were dynamically presented based on participants’ prior selections. Upon data collection, invalid responses were excluded according to the following pre-specified criteria: (a) uniform responses across all items; (b) completion time of less than 200 s; (c) logically inconsistent answers; and (d) blank responses to all open-ended questions.

### 2.5 Statistical analysis

Missing data were restricted to the four open-ended questions. As these items were optional and positioned at the end of the questionnaire, their missing responses were deemed Missing Completely at Random (MCAR). Accordingly, analyses about these questions were conducted only among respondents who provided answers. The number of excluded cases in each analysis was minimal and had a negligible impact on the overall results.

Data were managed in Excel and analyzed using SPSS 27.0. Normally distributed continuous variables are presented as mean ± standard deviation, non-normally distributed variables as median and interquartile range, and categorical data as frequency (percentage). Between-group differences were assessed using the chi-square test for categorical variables and one-way analysis of variance (one-way ANOVA) for continuous measures. Associations with key outcomes were examined through multiple linear regression and binary logistic regression, with results reported as regression coefficients (for linear regression), odds ratios (for logistic regression), and their respective 95% confidence intervals. A *p*-value < 0.05 was considered statistically significant.

## 3 Results

### 3.1 General information on respondents

The final sample consisted of 181 valid questionnaires out of 200 distributed, resulting in an effective response rate of 90.5%. Geographically, the respondents were predominantly from Central, North, and East China. The number of nurses per department exhibited a skewed distribution, with a median of 18 (IQR: 12–27) and a range from 2 to 260. Detailed demographic characteristics of the nursing staff are summarized (Table 2).

**Table 2:** General demographic information of nursing staff (*n* = 181).

Variables	Category	Frequency (persons)	Percentage (%)
Gender (*n* = 181)	Male	23	12.70
	Female	158	87.29
Age (*n* = 181)	< 25	59	32.59
	25–35	85	46.96
	36–45	31	17.12
	> 45	6	3.31
Education attainment (*n* = 181)	Bachelor’s degree	136	75.13
	Master’s degree	34	18.78
	Associate degree	8	4.41
	Doctoral degree	3	1.65
Hospital grade^a^ (*n* = 181)	Tertiary hospital	142	78.45
	Secondary hospital	20	11.05
	Primary hospital	19	10.49
Geographic distribution (*n* = 181)	Central China	67	37.1
	North China	48	26.52
	East China	43	23.76
	Southwest China	11	6.08
	South China	9	4.97
	Northwest China	3	1.66
Type of nurse (*n* = 181)	Nurse intern	29	16.02
	Staff nurse	139	76.79
	Nurse manager	13	7.18
Department^b^ (*n* = 152)	Surgery department	50	32.89
	Internal medicine department	36	23.68
	Primary healthcare institution	19	12.50
	Intensive care unit	17	11.18
	Outpatient and emergency department	16	10.52
	Other	14	9.21
Years of experience^c^ (*n* = 152)	<1 year	28	18.42
	1–5 years	53	34.84
	6–10 years	21	13.81
	> 10 years	50	32.89
Professional title^d^ (*n* = 152)	Junior	96	63.15
	Intermediate	50	32.89
	Senior	6	3.94

^a^China’s hospital system is categorized into three levels: tertiary, secondary, and primary hospitals. ^d^The Chinese nursing professional title system is structured into three categories: Junior, Intermediate, and Senior. The criteria for promotion include years of work experience and professional achievements. ^b, c, d^The total sample size is 152, as 29 nurse interns did not apply to these variables.

### 3.2 Description of the current status of the GAI application

Among the 181 nurses surveyed, 168 (92.81%) reported using GAI. Chi-square analysis revealed no significant differences in GAI usage across age, work experience, education level, or professional roles (all *p* > 0.05), as shown in Supplementary Table B-1. A binary logistic regression model further confirmed that none of these demographic variables were significant predictors of GAI adoption (all *p* > 0.05), as all odds ratios had confidence intervals including 1: age (OR = 1.113, 95% CI: 0.325–3.817), work experience (OR = 1.160, 95% CI: 0.495–2.719), education level (OR = 0.834, 95% CI: 0.242–2.882), and type of nurse (OR = 0.356, 95% CI: 0.077–1.657). These results indicate that GAI adoption among Chinese nurses is not limited to specific demographic subgroups, suggesting the involvement of broader influencing factors, shown in Supplementary Table B-2.

The frequency of GAI usage among nurses was distributed as follows: 35.12% (59) reported using it several times per week, 27.38% (46) occasionally, 21.43% (36) frequently, 11.31% (19) several times per month, and 4.76% (8) once daily. One-way ANOVA revealed significant differences in usage 4.76% (8) frequency by age (*F* = 3.317, *p* = 0.021) and work experience (*F* = 7.501, *p* < 0.001), as shown in Supplementary Table B-3. These findings were further supported by multiple linear regression, which identified work experience as a significant positive predictor of usage frequency (*B* = 0.507, *t* = 3.326, *p* = 0.001) in an overall statistically significant model (*F* = 6.322, *p* < 0.001), as shown in Supplementary Table B-4.

**Figure 1:**
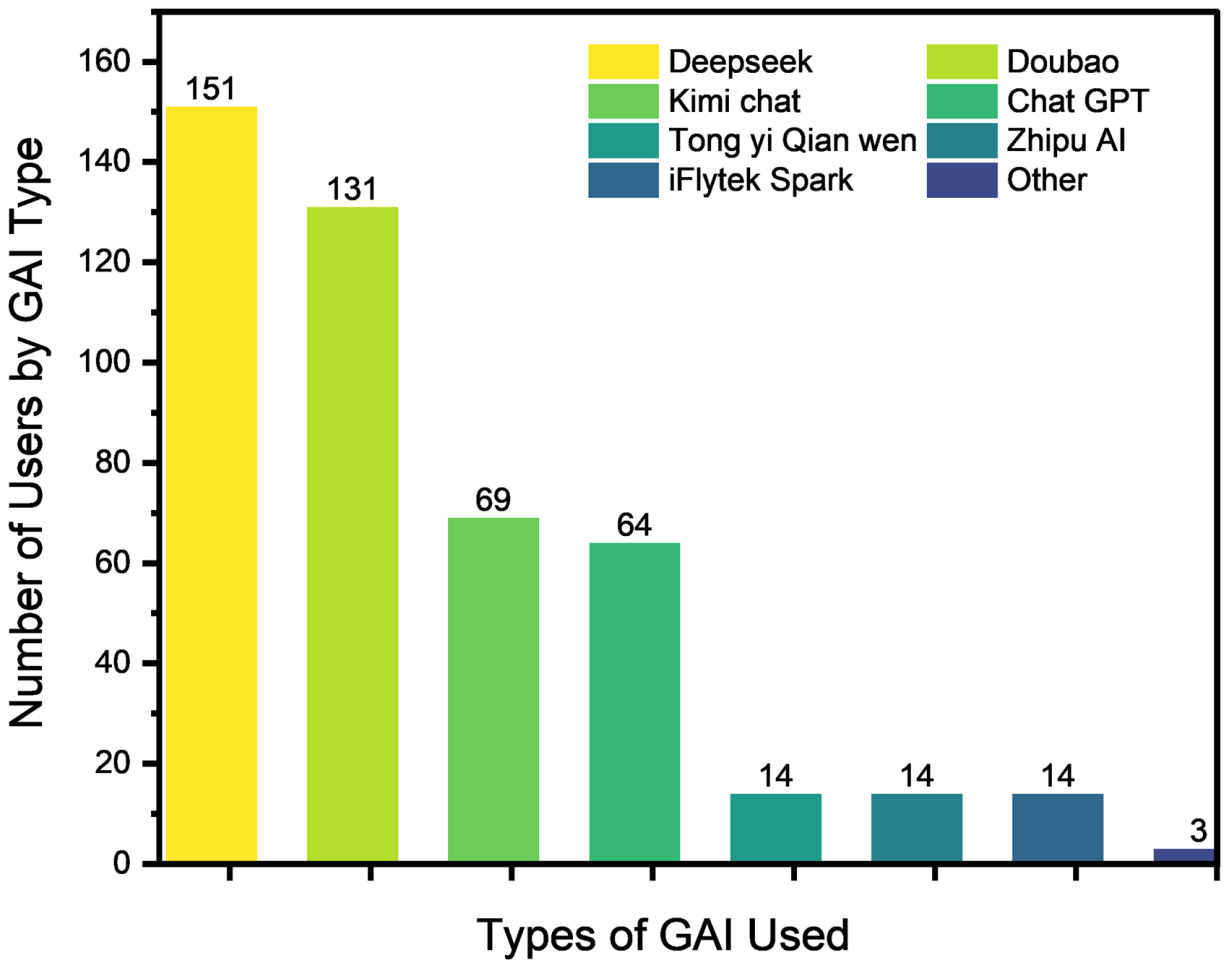
Describing the types of GAI usage.

Regarding the types of GAI used, non-domestic tools accounted for only 64 (13.91%) of the user base, whereas the local platforms DeepSeek and Doubao constituted 151 (32.83%) and 131 (28.48%) of users, respectively (Figure 1). This notable preference for domestic platforms likely reflects their enhanced Chinese language processing capabilities, fits more naturally into the digital tools nurses already use daily, and reduces accessibility barriers for users in China.

**Figure 2:**
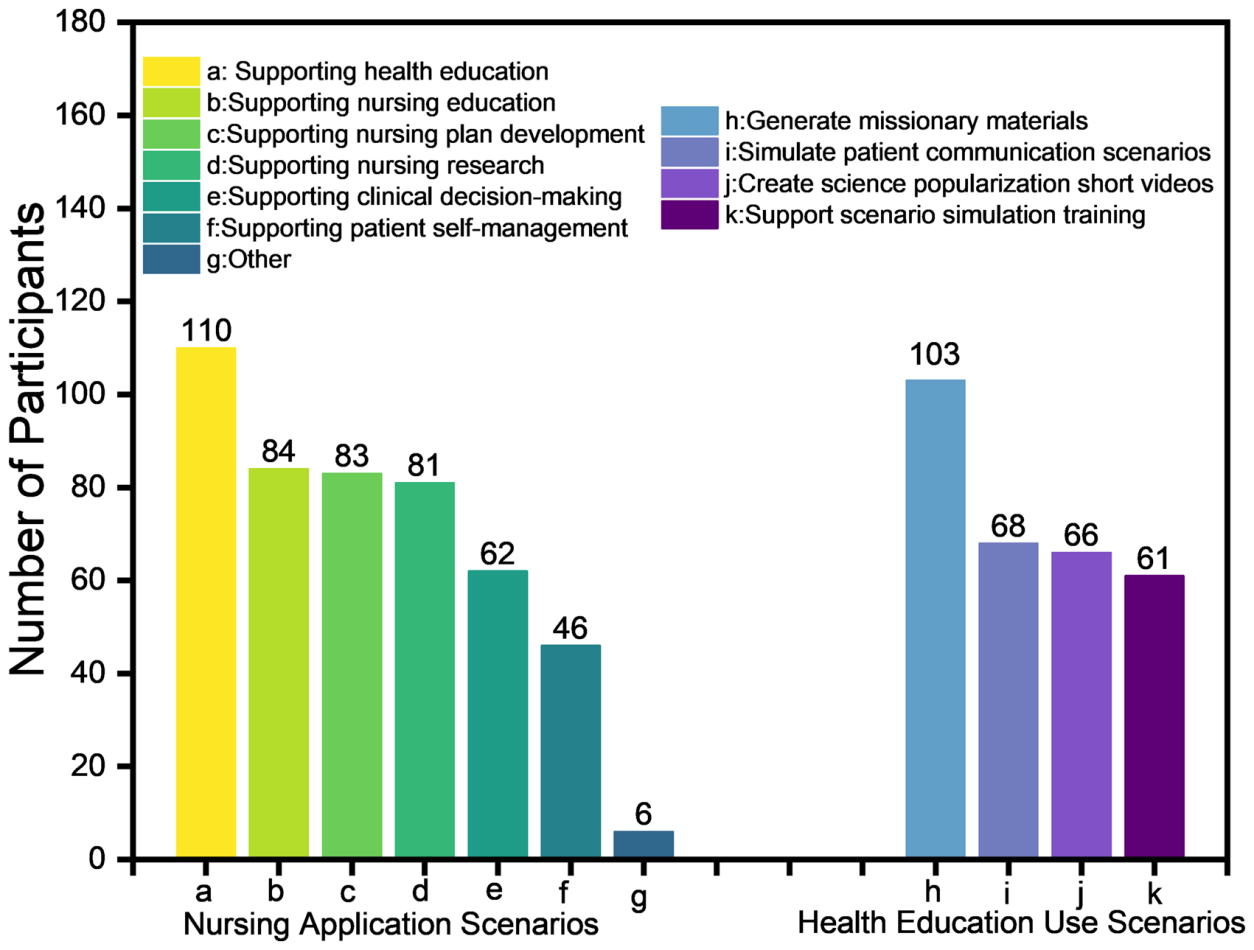
Descriptive summary of GAI functions across usage scenarios.

**Figure 3:**
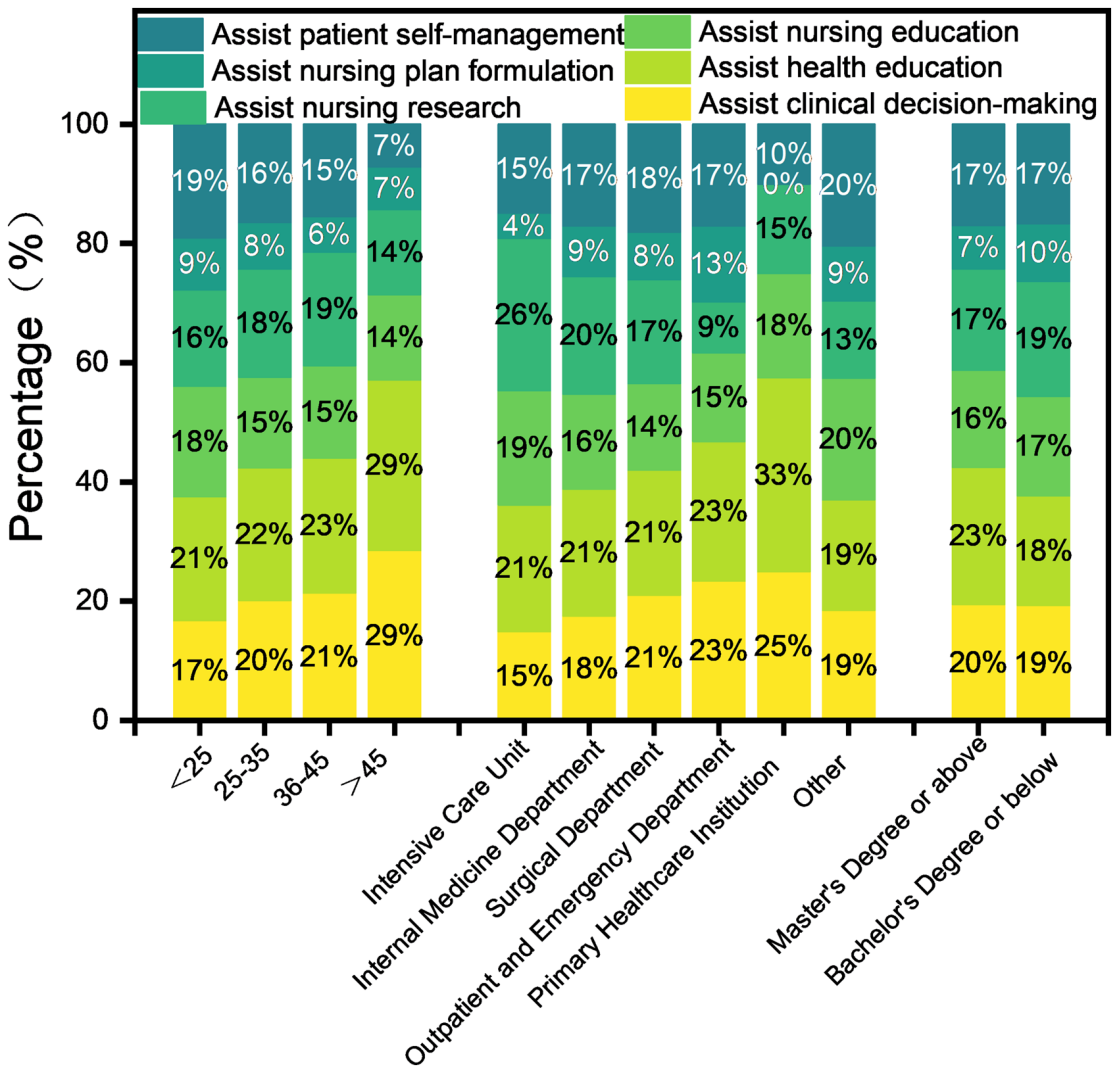
Descriptive overview of GAI use scenarios across education, department, and age groups.

In nursing application scenarios, 110 (65.48%) nurses employ GAI as a health education tool. Among these, 103 nurses (61.30%) used GAI to generate textual and graphic content for health education (Figure 2). This may be attributed to GAI’s ability to efficiently create patient education materials, thereby compensating for nurses’ time constraints. The usage scenarios of GAI across departments, age groups, and education levels are as shown in Figure 3.

An “Other” option was incorporated to encompass unanticipated scenarios; its uptake was negligible (e.g., only six participants selected it for GAI applications), and all open-text responses were devoid of substantive content.

### 3.3 Description of the current status of GAI use in departments

The survey on the importance of GAI in the all department showed 92 (50.82%) as average, 78 (43.08%) as important, and 11 (6.07%) as not important. The survey of the availability of training in GAI in the departments was 122 (67.4%) without training and 59 (32.6%) with training. As for whether the department used GAI techniques in clinical work, 75 (41.43%) did and 106 (58.57%) did not. The limited level of clinical application of GAI, along with the absence of a training system, reveals the challenges in transitioning the technology from awareness to clinical implementation, and further uncovers significant deficiencies in institutional support, professional capacity building, and deep integration with clinical scenarios.

### 3.4 Description of the evaluation and barriers to GAI

Survey results indicated that 72.39% of nurses perceived GAI outputs as accurate or very accurate, while 79.75% reported being satisfied or very satisfied with GAI. Univariate analysis revealed significant associations between perceived accuracy and education level (*F* = 6.977, *p* < 0.001) and between satisfaction and work experience (*F* = 3.835, *p* = 0.011), as shown in Supplementary Table B-3. Multiple linear regression further demonstrated that work experience significantly predicted lower satisfaction (*B* = –0.145, *p* = 0.026), while education level showed a marginally significant positive relationship with accuracy perceptions (*B* = 0.180, *p* = 0.050), as shown in supplementary material (Tables 3, 4). These findings suggest that higher education levels correlate with more favorable accuracy assessments, whereas greater clinical experience is associated with reduced satisfaction regarding GAI implementation.

**Table 3:** Demographic predictors of perceived accuracy in GAI use (*n* = 168).

Category	Unstandardized coefficients		Standardized coefficients	*t*	*p*	Collinearity diagnostics	
	*B*	Standard error	*Beta*			VIF	Tolerance
Constant	1.996	0.294	–	6.788	0.000***	–	–
Age	0.042	0.097	0.055	0.432	0.666	2.753	0.363
Years of experience	−0.083	0.065	−0.163	−1.265	0.208	2.882	0.347
Education attainment	0.180	0.091	0.156	1.973	0.050	1.083	0.923
Type of nurse	−0.037	0.110	−0.029	−0.338	0.736	1.267	0.789
*F*	*F* (4, 163) = 2.382, *p* = 0.054						
D-W value	1.789						

Dependent variable = Perceived accuracy of GAI in problem-solving. *p < 0.05, **p < 0.01, ***p < 0.001.

**Table 4:** Impact of general demographic characteristics on attitudes toward GAI use (*n* = 168).

**Category**	**Unstandardized coefficients**		**Standardized coefficients**	***t***	***p***	**Collinearity diagnostics**	
	***B***	**Standard error**	***Beta***			**VIF**	**Tolerance**
Constant	1.873	0.289	-	6.486	0.000***	-	-
Age	0.027	0.096	0.036	0.288	0.774	2.753	0.363
Years of experience	−0.145	0.064	−0.289	−2.250	0.026*	2.882	0.347
Education attainment	0.078	0.090	0.068	0.870	0.386	1.083	0.923
Type of nurse	0.199	0.108	0.157	1.844	0.067	1.267	0.789
*F*	*F* (4,163) = 3.045, *p* = 0.019						
D-W value	2.027						

Dependent variable = Attitudes toward GAI. *p < 0.05, **p < 0.01, ***p < 0.001.

**Figure 4:**
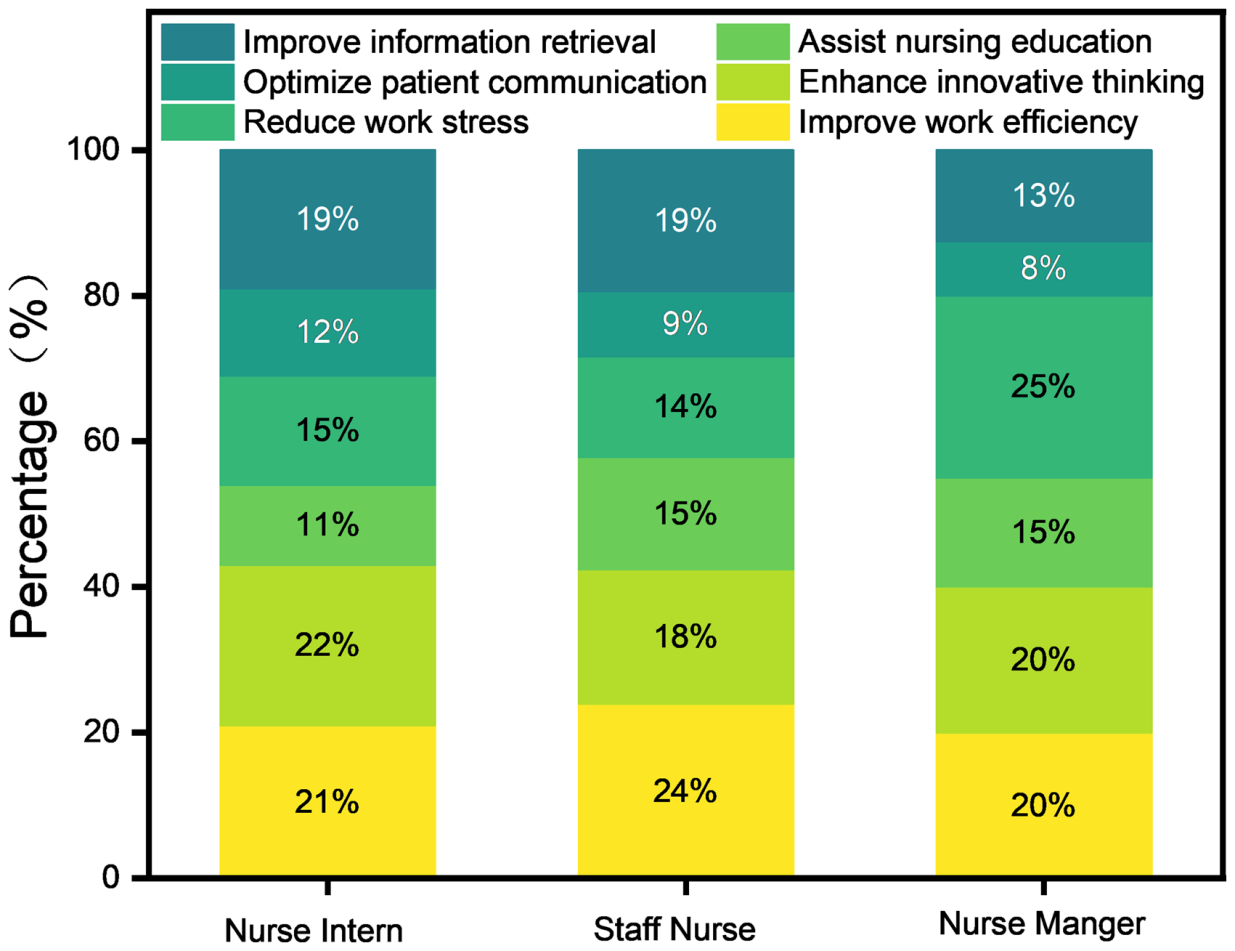
Descriptive profile of GAI benefits across caregiving roles.

The perceived benefits of GAI demonstrated role-specific patterns across nursing positions. Nurse managers reported primarily reduced work pressure, staff nurses emphasized improved work efficiency, and nurse interns highlighted enhanced innovative thinking (Figure 4). This functionally specialized assistance aligns with their distinct workflow priorities and task demands: managers benefit from administrative load reduction, clinical nurses from optimized task execution, and interns from expanded learning and innovation opportunities.

Survey results identified several key challenges in GAI adoption among nurses (Table 5). The most prevalent challenge was unfamiliarity with how to formulate effective instructions for AI tools (27.11%), which is consistent with the reported lack of adequate GAI training by respondents. Concerns about the potential erosion of clinical autonomy (26.61%) primarily stemmed from underlying reliability concerns regarding GAI outputs, underscoring the necessity of human-supervised implementation frameworks. Training in clinical application skills represented the most urgently expressed need (20.65%), with no respondents selecting the “Other” category.

**Table 5:** Problems and concerns with the use of GAIs (*n* = 181).

**Problem**	**Item**	**Frequency (persons)**	**Percentage (%)**
1. What are some of the main problems you have encountered with GAI? ^a^(*n* = 168)	Lack of familiarity with the generate command	93	27.11
	Inadequate understanding of system features	80	23.32
	Slow system response time	59	17.20
	Lack of credibility in generated results	50	14.58
	Low patient trust in GAI-generated content	40	11.66
	Complexity of operation	21	6.12
2. What are your concerns about GAI? (*n* = 181)	Impact on autonomy in clinical decision-making	120	26.61
	Concerns over patient privacy breaches	81	17.96
	Ethical conflicts and value misalignment	76	16.85
	Challenges in accountability attribution	70	15.52
	Insufficiency of emotional support in nurse–patient interactions	59	13.08
	Risk of role substitution in the nursing profession	45	9.98
3. If you could be trained in GAI techniques, what are the areas of interest? (*n* = 181)	Clinical practice training	115	20.65
	Nursing research training	111	19.93
	Training in home care for assisted patients	97	17.41
	Basic Operations and Ethics Training	97	17.41
	Training in the application of the care management system	73	13.11
	Applied training in nursing education	64	11.49

The percentages represent the constituent ratio. The questions in this table are multiple-choice questions. ^a^As 13 participants reported no prior experience with GAI, they were excluded from the analysis of this section, resulting in an analytical sample size of n = 168 for this question.

### 3.5 Description of nursing staff’s functional needs, evaluation, and technical recommendations for GAI

Analysis of functional needs revealed that clinical operational support was the most frequently requested GAI function (25.9%). Improving work efficiency was the most commonly recognized benefit (55.19%). However, concerns about insufficient clinical accuracy represented the most frequently cited barrier (40.96%), while concerns about data security were also commonly expressed. For future development, 55.64% of nursing staff recommended developing GAI as an intelligent, evidence-based nursing assistant (Table 6). These findings collectively indicate a practical demand for and generally positive reception of GAI, while underscoring persistent challenges regarding clinical accuracy.

**Table 6:** Functions that caregivers would like to have in the GAI and evaluation and recommendations for their use (*n* = 181).

**Problem**	**Category**	**Item**	**Frequency (persons)**	**Percentage (%)**
1. Which GAI capabilities are most needed in clinical nursing work? (*n* = 164)^a^	Clinical operations support	GAI assistant for basic care operations	22	25.90
		GAI-based clinical risk monitoring	9	
		GAI-facilitated nurse–patient communication	7	
		GAI-guided clinical workflow development	5	
	Expertise support	Intelligent Nursing Knowledge Update System	25	19.27
		Nursing Innovation Support Platform	4	
		Anthropomorphic Intelligent Thinking Engine	3	
	Clinical decision support	Smart Planning for Nursing	7	18.67
		Clinical Auxiliary Diagnostic Support	10	
		Evidence-based nursing decision support	14	
	Instrumental support	Intelligent Nursing Document Generation	11	14.45
		Intelligent PPT, image generation	5	
		Intelligent generation of health education videos	8	
	Research support	GAI-powered writing assistant	13	9.03
		GAI-facilitated literature analysis	2	
	health promotion	Personalized health promotion	13	7.83
	Emotional support	Emotional support capabilities	5	4.81
		Patient-centered communication facilitation	3	
2. What do you think are the advantages of GAI in nursing? (*n* = 177)^b^	Enhancement of clinical productivity	Enhanced usability	32	55.19
		Improved operational efficiency	69	
	Intelligent knowledge support systems	Intelligent Information Panorama	24	16.94
		Intelligent precision cognition	7	
	Intelligent assistance to reduce the burden	Intelligent clinical burden reduction	21	14.21
		Intelligent labor cost optimization	5	
	Intelligent efficiency gains in quality of care	Developing innovative thinking	9	8.74
		Decision support	7	
	No advantage		9	4.92
3. What do you think are the shortcomings of GAI in nursing? (*n* = 166)^c^	Low clinical accuracy	Insufficient accuracy of information	39	40.96
		Insufficient professionalism	29	
	Lack of humanized services	Lack of empathetic response	16	16.26
		Depersonalization of care	11	
	Technological immaturity	Limited functionality and inefficiency	12	15.66
		Weak emergency decision-making capacity	3	
		Mechanical rigidity and lack of trust	11	
	Application maturity	Limitations in application and difficulty in popularization	8	13.85
		Weak autonomous judgment	8	
		Data security risks	7	
	Adequate		22	13.25
4. What are your suggestions for GAI? (*n* = 133)^d^	Intelligent, evidence-based nursing assistant	Providing evidence-based precision responses	33	55.64
		Increase specialization and adaptability	28	
		Expanding the authoritative medical knowledge base	8	
		Provide concise, easy-to-understand explanations	5	
	Standardized training and smart upgrades	Standardized training in the use of GAI	10	31.58
		Popularization of science and dissemination of values	9	
		Technology development and performance breakthrough	23	
	Intelligent care interaction with humanization	Warm intelligent interaction	6	9.77
		Humanized communication	6	
	Intelligent privacy protection	Protection of privacy	5	3.76

The percentages represent the constituent ratio. The items in this table are open-ended questions. This table presents data from open-ended questions designed to capture spontaneous perspectives. Therefore, some survey respondents provided more than one answer, and some chose not to respond. ^a^There were 164 respondents to this question, providing a total of 166 responses. ^b^There were 177 respondents to this question, providing a total of 183 responses. ^c^There were 166 respondents to this question, providing a total of 166 responses. ^d^ There were 133 respondents to this question, providing a total of 133 responses.

## 4 Discussion

### 4.1 The application of GAI among a sample of Chinese nurses: high popularity and low frequency of use

The survey revealed a high adoption rate of GAI among the sampled Chinese nursing staff, indicating widespread adoption. However, usage frequency remained generally low, with only 26.19% of respondents reporting daily or more frequent use. Regression analysis identified work experience as a significant positive predictor of usage frequency (*B* = 0.507, *p* = 0.001), suggesting greater integration into daily workflows among experienced nurses, consistent with findings reported by Kang et al. (24). The most widely used GAI platforms were DeepSeek and Doubao. According to Wu (25), the broad adoption of DeepSeek across multiple fields can be attributed to its high-performance yet low-cost training paradigm, robust on-premises deployment capability, and commitment to open-source collaboration.

Our findings reveal a distinct emphasis compared to those of Biswas and Castonguay (26, 27). While their studies highlighted AI applications in nursing education and documentation automation, our research—situated within China’s unique healthcare landscape—identifies health education as the dominant application area for Generative AI. This divergence likely reflects differences in nursing informatics infrastructure maturity and region-specific priorities, particularly China’s emphasis on preventive care. Powered by its robust natural language processing and content generation capabilities, GAI is progressively transforming traditional nursing workflows (28). It generates diverse simulated cases for training (28), utilizes speech recognition to facilitate structured documentation and improve efficiency (29), and rapidly produces personalized health guidance materials to support scalable, precise patient education and preventive care (30).

In conclusion, while GAI adoption is common among Chinese nurses, the depth and frequency of its use require enhancement. To fully realize its potential, we recommend targeted training programs, optimized functional design, and the expansion of application scenarios into broader aspects of clinical practice.

### 4.2 The degree of importance attached to GAI by the relevant departments needs to be improved

The study found that about 56% of nursing staff reported that their departments paid limited or no attention to GAI, suggesting that organizational recognition and support for GAI need to be strengthened. This limited organizational engagement may be related to current constraints in GAI’s clinical application. For instance, Shoja et al. (31) reported that GAI achieved only 71.7% accuracy in clinical decision-making tasks, with notable risks including the omission of critical clinical details and the generation of inaccurate information, particularly in complex cases.

In terms of training provision, only 32.6% of departments in this study reported having conducted GAI-related training. This proportion is lower than that reported by Chen (32), possibly reflecting differences in hospital levels and regional technological resources across studies. This gap highlights the importance of healthcare organizations collaborating with educational institutions to develop competency-based curricula that enhance nurses’ AI literacy, including skills in critically evaluating AI outputs and the ability to adhere to ethical guidelines in practice.

Based on these findings, healthcare organizations should strengthen institutional support and establish systematic training programs. Such initiatives are essential to bridge existing gaps and effectively leverage GAI’s potential in alleviating nursing workload, enhancing service quality and operational efficiency, and thereby improving patient satisfaction.

### 4.3 Coexistence of high satisfaction and deep concern: the overall appraisal of GAI among surveyed Chinese nurses

Most nurses considered GAI outputs satisfactory and accurate, consistent with reports by Noy and Chan (33, 34). However, regression analysis indicated that higher education levels marginally predicted more positive accuracy perceptions (*B* = 0.180, *p* = 0.050), while greater clinical experience significantly predicted lower satisfaction (*B* = –0.145, *p* = 0.026). This divergence suggests that although nurses generally acknowledge GAI’s technical capabilities, those with extensive clinical experience maintain more critical perspectives regarding its practical implementation, potentially reflecting concerns about its integration into complex clinical workflows. Unlike prior research on overall AI attitudes (35), few studies have systematically examined attitude differences based on years of clinical experience-an area that warrants further investigation.

However, during the application process, nurses encounter a number of issues and worries. Among these issues, prominent issues included unfamiliarity with generated instructions and poor comprehension of GAI functions, consistent with Ali (36), who found that over 50% of caregivers lacked systematic knowledge of AI basics, applications, and operations. Major concerns involved loss of clinical autonomy, potential privacy breaches, and inaccurate information. Chen and Rony (37, 38) indicated that GAI training relies on extensive patient data, raising privacy risks from model queries. Rony (38) also noted that nurses often worry about human-computer tension and constrained decision-making autonomy. In terms of training needs, nursing staff expressed strong demand for GAI education, particularly in clinical practice and nursing research, echoing Rony’s (39) findings.

To support the effective integration of GAI in nursing, multi-stakeholder collaboration is essential: nursing managers should formulate guidelines and launch pilot programs to clarify nurses’ roles in reviewing GAI output; nursing educators should foster students’ critical use of GAI; and developers should improve model professionalism, optimize workflow integration, and enhance data security. These coordinated efforts will help achieve safe and effective GAI deployment to empower nursing practice.

Based on these findings, while GAI demonstrates considerable adoption potential and is generally perceived as accurate and satisfactory among Chinese nurses, its effective integration into clinical practice requires addressing critical concerns regarding operational proficiency, clinical reliability, and workflow compatibility through coordinated multi-stakeholder efforts.

### 4.4 Clinical needs of GAI for nursing staff and advantages and limitations of its application

While GAI holds promise for nursing, most current research focuses on its applications, with few studies investigating caregivers’ actual needs. In this study, clinical operational assistance was the most anticipated application of GAI among nursing staff, aligning with Pepito et al. (40). This may be related to China’s high job demands, work pressure, resource constraints, and nursing shortages (41). Respondents identified improved clinical efficiency as the primary benefit of GAI, consistent with Nova et al. (42). However, key limitations were identified, particularly concerning clinical accuracy and data security—issues also emphasized by Almaghaslashi et al. (43). Furthermore, nurses provided constructive suggestions for GAI development, emphasizing the hope that it evolves into a knowledgeable, evidence-based assistant capable of providing clinical decision support grounded in reliable professional data. These findings differ from those of Martin-Hammond et al. (44, 45), who reported that nurses expected AI to aid mainly in risk recognition and routine task management. These discrepancies may be explained by variations in sample characteristics, research settings, or differences in nurses’ AI awareness.

To address low clinical accuracy and data security, it is crucial to establish robust data protection and clinical review mechanisms within healthcare organizations. Concurrently, policymakers may consider advancing specialized legislation and tool certification. Such coordinated efforts will be instrumental in ensuring the compliant and safe implementation of GAI.

In summary, GAI introduces new perspectives to nursing. Nursing staff should fully recognize the advantages and limitations of GAI and take the initiative to integrate and optimize its use in practice. Meanwhile, researchers and developers should strive to enhance the clinical applicability and accuracy of AI tools to address the core demands of nursing practice effectively.

## 5 Conclusion

This study reveals widespread but superficial adoption of Generative AI among Chinese nurses, characterized by dominant use of local platforms and primary application in health education. We identified role-specific benefits—reducing administrative pressure for managers, improving efficiency for staff nurses, and stimulating innovation for interns. Major barriers include concerns over professional accuracy and clinical autonomy. Future development should focus on enhancing clinical accuracy and providing systematic training to facilitate GAI’s evolution into an evidence-based nursing assistant.

## 6 Limitations and future directions

This study has several limitations. First, the survey instrument was developed specifically for this investigation due to the lack of validated scales in this emerging field. While content validity was established through expert review, the instrument has not undergone comprehensive psychometric validation, which may influence the interpretation of its constructs. Second, the use of a convenience sampling approach, though practical, may introduce self-selection bias, potentially overrepresenting nurses already interested in digital technologies. Although the sample reflected diversity in hospital levels, regions, and roles, it is not statistically representative of the national nursing population. Third, the analysis is primarily descriptive, which precludes causal inferences regarding certain relationships among some variables. Finally, as a cross-sectional design, this study cannot assess the evolution of attitudes or long-term integration of GAI tools, underscoring the need for longitudinal or mixed-methods approaches in future research. Despite these limitations, the methodological choices were appropriate for this exploratory study, which provides a foundational evidence base for subsequent confirmatory research.

## Data Availability

The original contributions presented in the study are included in the article/supplementary material, further inquiries can be directed to the corresponding author/s.

## References

[1] Banh L Strobel G. Generative artificial intelligence. Electron Mark. (2023) 33:63. doi: 10.1007/s12525-023-00680-1

[2] Javanmard S. Revolutionizing medical practice: the impact of artificial intelligence (AI) on healthcare. Open Access J Appl Sci Technol. (2024) 2:07. doi: 10.33140/OAJAST.02.01.07

[3] Rabbani SA El-Tanani M Sharma S Rabbani SS El-Tanani Y Kumar R . Generative artificial intelligence in health care: applications, implementation challenges, and future directions. BioMedInformatics. (2025) 5:37. doi: 10.3390/biomedinformatics5030037

[4] Huong XV. The implications of artificial intelligence for educational systems: challenges, opportunities, and transformative potential. Am J Soc Sci Educ Innov. (2024) 6:101–11. doi: 10.37547/tajssei/Volume06Issue03-17

[5] Ronquillo CE Peltonen L Pruinelli L Chu CH Bakken S Beduschi A . Artificial intelligence in nursing: priorities and opportunities from an international invitational think-tank of the nursing and artificial intelligence leadership collaborative. J Adv Nurs. (2021) 77:3707–17. doi: 10.1111/jan.14855, 34003504 PMC7612744

[6] Pailaha AD. The impact and issues of artificial intelligence in nursing science and healthcare settings. SAGE Open Nurs. (2023) 9:23779608231196847. doi: 10.1177/23779608231196847, 37691725 PMC10492460

[7] Johnson KB Wei W Weeraratne D Frisse ME Misulis K Rhee K . Precision medicine, AI, and the future of personalized health care. Clin Transl Sci. (2020) 14:86–93. doi: 10.1111/cts12884 32961010 PMC7877825

[8] Kuwaiti AA Nazer K Al-Reedy A Al-Shehri S Al-Muhanna A Subbarayalu AV . A review of the role of artificial intelligence in healthcare. J Pers Med. (2023) 13:951. doi: 10.3390/jpm13060951, 37373940 PMC10301994

[9] Martinez-Ortigosa A Martinez-Granados A Gil-Hernández E Rodriguez-Arrastia M Ropero-Padilla C Roman P. Applications of artificial intelligence in nursing care: a systematic review. J Nurs Manag. (2023) 2023:3219127. doi: 10.1155/2023/3219127, 40225652 PMC11919018

[10] Templin T Perez MW Sylvia S Leek J Sinnott-Armstrong N. Addressing 6 challenges in generative AI for digital health: a scoping review. PLOS Digit Health. (2024) 3:e0000503. doi: 10.1371/journal.pdig.0000503, 38781686 PMC11115971

[11] Reddy S. Generative AI in healthcare: an implementation science informed translational path on application, integration and governance. Implement Sci. (2024) 19:27. doi: 10.1186/s13012-024-01357-9, 38491544 PMC10941464

[12] Sommer D Schmidbauer L Wahl F. Nurses' perceptions, experience and knowledge regarding artificial intelligence: results from a cross-sectional online survey in Germany. BMC Nurs. (2024) 23:205. doi: 10.1186/s12912-024-01884-2, 38539169 PMC10967047

[13] Al Omari O Alshammari M Al Jabri W Al Yahyaei A Aljohani KA Sanad HM . Demographic factors, knowledge, attitude and perception and their association with nursing students' intention to use artificial intelligence (AI): a multicentre survey across 10 Arab countries. BMC Med Educ. (2024) 24:1456. doi: 10.1186/s12909-024-06452-5, 39696341 PMC11653676

[14] Von Gerich H Moen H Block LJ Chu CH DeForest H Hobensack M . Artificial intelligence-based technologies in nursing: a scoping literature review of the evidence. Int J Nurs Stud. (2022) 127:104153. doi: 10.1016/j.ijnurstu.2021.104153, 35092870

[15] O’Connor S Yan Y Thilo FJS Felzmann H Dowding D Lee JJ. Artificial intelligence in nursing and midwifery: a systematic review. J Clin Nurs. (2022) 32:3311–43. doi: 10.1111/jocn.16478 35908207

[16] Seibert K Domhoff D Bruch D Schulte-Althoff M Fürstenau D Biessmann F . Application scenarios for artificial intelligence in nursing care: rapid review. J Med Internet Res. (2021) 23:e26522. doi: 10.2196/26522, 34847057 PMC8669587

[17] King DR Nanda G Stoddard J Dempsey A Hergert S Shore JH . An introduction to generative artificial intelligence in mental health care: considerations and guidance. Curr Psychiatry Rep. (2023) 25:839–46. doi: 10.1007/s11920-023-01477-x, 38032442

[18] Wang Y Li N Chen L Wu M Meng S Dai Z . Correction: guidelines, consensus statements, and standards for the use of artificial intelligence in medicine: systematic review. J Med Internet Res. (2023) 25:e55596. doi: 10.2196/55596, 38128080 PMC10763555

[19] Kurniawan MH Handiyani H Nuraini T Hariyati RTS Sutrisno S. A systematic review of artificial intelligence-powered (AI-powered) chatbot interventions for managing chronic illness. Ann Med. (2024) 56:2316903. doi: 10.1080/07853890.2024.2302980, 38466897 PMC10930147

[20] Shi J Mo X Sun Z. Application of content validity index in scale development. J Cent South Univ (Med Sci). (2012) 37:152–5. doi: 10.3969/j.issn.1672-7347.2012.02.007 22561427

[21] Davis LL. Instrument review: getting the most from your panel of experts. Appl Nurs Res. (1992) 5:194–7. doi: 10.1016/s0897-1897(05)80008-4

[22] Waltz CF Strickland OL Lenz ER. Measurement in nursing and health research. 3rd ed. New York: Springer (2005). 157 p.

[23] Kendall MG Stuart A. The advanced theory of statistics, vol. 2. 4th ed. London: Charles Griffin & Company Ltd (1979).

[24] Kang R Xuan Z Tong L Wang Y Jin S Xiao Q. Nurse researchers' experiences and perceptions of generative AI: qualitative semistructured interview study. J Med Internet Res. (2025) 27:e65523. doi: 10.2196/65523, 40853413 PMC12377238

[25] Wu P Zhu Y. A timely quick literature review on the DeepSeek in Chinese publication. Chin Stud Mon. (2025) 3:14–21. doi: 10.70731/0h00wq42

[26] Biswas A Talukdar W. Intelligent clinical documentation: harnessing generative AI for patient-centric clinical note generation. Int J Innov Sci Res Technol. (2024) 9:994–1008. doi: 10.38124/ijisrt/ijisrt24may1483

[27] Castonguay A Farthing P Davies S Vogelsang L Kleib M Risling T . Revolutionizing nursing education through AI integration: a reflection on the disruptive impact of ChatGPT. Nurse Educ Today. (2023) 129:105916. doi: 10.1016/j.nedt.2023.105916, 37515957

[28] Brügge E Ricchizzi S Arenbeck M Zoller C Welz A Lauerer J . Large language models improve clinical decision making of medical students through patient simulation and structured feedback: a randomized controlled trial. BMC Med Educ. (2024) 24:1391. doi: 10.1186/s12909-024-06399-7 39609823 PMC11605890

[29] Lee TY Li CC Chou KR Chung MH Hsiao ST Guo SL . Machine learning-based speech recognition system for nursing documentation: a pilot study. Int J Med Inform. (2023) 178:105213. doi: 10.1016/j.ijmedinf.2023.105213, 37690224

[30] Aydin S Karabacak M Vlachos V Margetis K. Large language models in patient education: a scoping review of applications in medicine. Front Med. (2024) 11:1477898. doi: 10.3389/fmed.2024.1477898, 39534227 PMC11554522

[31] Shoja MMM Monica Rajput V. The emerging role of generative artificial intelligence in medical education, research, and practice. Cureus. (2023) 15:e40883. doi: 10.7759/cureus.40883 37492829 PMC10363933

[32] Chen X Ryoo J. Advancing AI in healthcare through professional training: insights from Chinese practitioners. Sci Technol Sci Soc. (2025) 2:95–110. doi: 10.59324/stss.2025.2(1).08

[33] Noy S Zhang W. Experimental evidence on the productivity effects of generative artificial intelligence. Science. (2023) 381:187–92. doi: 10.1126/science.Adh2586, 37440646

[34] Chan CKY Hu W. Students' voices on generative AI: perceptions, benefits, and challenges in higher education. Int J Educ Technol High Educ. (2023) 20:43. doi: 10.1186/s41239-023-00411-8

[35] Alruwaili MM Abuadas FH Alsadi M Alruwaili AN Elsayed Ramadan OM Shaban M . Exploring nurses' awareness and attitudes toward artificial intelligence: implications for nursing practice. Digit Health. (2024) 10:20552076241271803. doi: 10.1177/20552076241271803, 39114115 PMC11304479

[36] Ali FN. Artificial intelligence in nursing practice: challenges and barriers. Helwan Int J Nurs Res Pract. (2024) 3:217–30. doi: 10.21608/hijnrp.2024.292923.1169

[37] Chen Y Esmaeilzadeh P. Generative AI in medical practice: in-depth exploration of privacy and security challenges. J Med Internet Res. (2024) 26:e53008. doi: 10.2196/53008, 38457208 PMC10960211

[38] Rony MKK Numan SM Akter K Tushar H Debnath M Johra FT . Nurses' perspectives on privacy and ethical concerns regarding artificial intelligence adoption in healthcare. Heliyon. (2024) 10:e40016. doi: 10.1016/j.heliyon.2024.e40016 39281626 PMC11400963

[39] Rony MKK Kayesh I Bala SD Akter F Parvin MR. Artificial intelligence in future nursing care: exploring perspectives of nursing professionals—a descriptive qualitative study. Heliyon. (2024) 10:e25718. doi: 10.1016/j.Heliyon.2024.e25718, 38370178 PMC10869862

[40] Pepito JA Locsin R. Can nurses remain relevant in a technologically advanced future? Int J Nurs Sci. (2019) 6:106–10. doi: 10.1016/j.ijnss.2018.09.013, 31406875 PMC6608671

[41] Wang H, Liu Y, Zeng T, Wang Y, Li M, Yu X An international comparative study on nurse staffing policies: a scoping review. Chin J Nurs (2022) 57:2674–2682. Doi:doi: 10.3761/j.issn.0254-1769.2022.21.018

[42] Nova K. Generative AI in healthcare: advancements in electronic health records, facilitating medical languages, and personalized patient care. J Adv Anal Healthc Manag. (2023) 7:115–31.

[43] Almagazzachi A Mustafa A Sedeh AE AEV G Polianovskaia A Abood M . Generative artificial intelligence in patient education: ChatGPT takes on hypertension questions. Cureus. (2024) 16:e53441. doi: 10.7759/cureus.53441 38435177 PMC10909311

[44] Martin-Hammond A Vemireddy S Rao K. Exploring older adults’ beliefs about the use of intelligent assistants for consumer health information management: a participatory design study. JMIR Aging. (2019) 2:e15381. doi: 10.2196/15381, 31825322 PMC6931054

[45] Korchut A Szklener S Abdelnour C Tantinya N Hernández-Farigola J Ribes JC . Challenges for service robots—requirements of elderly adults with cognitive impairments. Front Neurol. (2017) 8:228. doi: 10.3389/fneur.2017.00228, 28620342 PMC5451499

