# Supplementary material for: Exploring the application of generative artificial intelligence in nursing: a cross-sectional study
Source: Front Public Health. 2026 Jan 28;14:1689418. doi: 10.3389/fpubh.2026.1689418 (PMC12893205; doi:10.3389/fpubh.2026.1689418)
Supplement: Supplementary file 1 [file Data_Sheet_1.pdf]

Appendix A:

Exploring the Application of Generative Artificial Intelligence in Nursing: A Questionnaire Survey

Dear Nursing Colleagues,

Thank you for participating in this survey. This questionnaire aims to investigate the application of Generative Artificial Intelligence (GAI) in the nursing field. Your feedback will provide valuable insights for the intelligent development of the nursing industry. This survey is anonymous, and the data will be used solely for academic research purposes. It will take approximately 3-5 minutes to complete. Please answer all questions based on your actual situation. The formats include single-choice, multiple-choice, fill-in-the-blank, and open-ended questions. There are no right or wrong answers. By proceeding with the survey, you indicate your voluntary consent to participate.

Part 1: Demographic Information

1. Name of your healthcare institution: ________ **(Fill-in-the-blank question)**

2. Level of your healthcare institution **(Multiple-choice question)**

A. Tertiary hospital

B. Secondary hospital

C. Primary hospital

D. Other (Please specify): ________

3. Your department: ________ **(Fill-in-the-blank question)**

4. Number of nurses in your department: ________ **(Fill-in-the-blank question)**

5. Your age: **(Single-choice question**）

A. < 25 years old

B. 25 - 35 years old

C.36 - 45 years old

D. > 45 years old

6. Your gender: **(Single-choice question)**

A. Male

B. Female

7. Your years of work experience: **(Single-choice question)**

A. < 1 year

B. 1 - 5 years

C. 6 - 10 years

D. > 10 years

8. Your highest educational qualification: **(Single-choice question)**

A. Associate degree

B. Bachelor's degree

C. Master's degree

D. Doctoral degree

9. Your professional title: **(Single-choice question)**

A. Junior nurse

B. Intermediate nurse

C. Senior nurse

10. Your position: **(Single-choice question)**

A. Nurse intern

B. Staff nurse

C. Nurse manager

Part 2: Current Personal Use of Generative AI

1. Have you ever used Generative AI? **(Single-choice question)**

A. Yes

B. No

2. How frequently do you use Generative AI? **(Single-choice question)**

A. Multiple times per day

B. Once per day

C. A few times per week

D. A few times per month

E. Occasionally

3. Which of the following Generative AI tools have you used? **(Multiple-choice question)**

A. ChatGPT

B. DeepSeek

C. Kimi Chat

D. Doubao

E. Tongyi Qianwen((GLM))

F. Zhipu AI

G. iFlytek Spark

H. Other (Please specify): ________

4. In which of the following scenarios do you use Generative AI? **(Multiple-choice question)**

A. Assisting clinical decision-making

B. Assisting in formulating nursing care plans

C. Assisting patient self-management

D. Assisting health education

E. Assisting nursing research

F. Assisting nursing education

G. Other (Please specify): ________

5. When using GAI to assist clinical decision-making, what are its primary functions? **(Multiple-choice question) (Displayed only if "Assisting clinical decision-making" is selected in Q4)**

A. Generating nursing differential diagnoses

B. Flagging high-risk patients

C. Recommending evidence-based nursing interventions

D. Interpreting the clinical significance of test/lab results

6. When using GAI to assist in formulating nursing care plans, in which specific aspects is it applied? **(Multiple-choice question) (Displayed only if "Assisting in formulating nursing care plans" is selected in Q4)**

A. Generating personalized care plans

B. Adjusting plan priority sequencing

C. Periodically adjusting care plans

D. Providing feedback on care plan implementation outcomes

7. When using GAI to assist patient self-management, in which specific aspects is it applied? **(Multiple-choice question) (Displayed only if "Assisting patient self-management" is selected in Q4)**

A. Creating personalized chronic disease management plans

B. Providing intelligent medication reminders and guidance

C. Developing rehabilitation training plans

D. Mental health screening and personalized intervention suggestions

8. When using GAI to assist health education, in which specific aspects is it applied? **(Multiple-choice question) (Displayed only if "Assisting health education" is selected in Q4)**

A. Generating illustrated educational materials

B. Assisting situational simulation training

C. Generating patient Q&A scenario simulations

D. Creating short health science videos

9. When using GAI to assist nursing research, in which specific aspects is it applied? **(Multiple-choice question) (Displayed only if "Assisting nursing research" is selected in Q4)**

A. Assisting in analyzing research hotspots and providing topic suggestions

B. Assisting in optimizing academic writing and language expression

C. Assisting with statistical analysis

D. Assisting in standardizing manuscript formatting

10. When using GAI to assist nursing education, in which specific aspects is it applied? **(Multiple-choice question) (Displayed only if "Assisting nursing education" is selected in Q4)**

A. Developing virtual simulation teaching cases

B. Optimizing nursing teaching program design

C. Generating nursing operational skill training programs

D. Designing assessment systems and intelligent grading

11. Please specify in which other scenarios you apply Generative AI: ________ **(Fill-in-the-blank question) (Displayed only if "Other" is selected in Q4)**

Part 3: Current Use of Generative AI in Your Department

1. How would you rate the level of importance your department places on Generative AI technology? **(Single-choice question)**

A. Very important

B. Important

C. Neutral

D. Unimportant

E. Very unimportant

2. Does your department provide any training related to Generative AI technology? **(Single-choice question)**

A. Yes

B. No

3. Is Generative AI technology applied in clinical work within your department? **(Single-choice question)**

A. Yes

B. No

Part 4: Evaluation of and Barriers to Using Generative AI

1. What benefits do you believe Generative AI offers you? **(Multiple-choice question)**

A. Improves work efficiency

B. Enhances innovative thinking

C. Assists clinical decision-making

D. Reduces work pressure

E. Optimizes patient communication

F. Increases information retrieval efficiency

G. Other (Please specify): ________

2. How would you rate the accuracy of Generative AI in solving problems? **(Single-choice question)**

A. Very accurate

B. Accurate

C. Neutral

D. Inaccurate

E. Very inaccurate

3. What is your overall satisfaction with Generative AI? **(Single-choice question)**

A. Very satisfied

B. Satisfied

C. Neutral

D. Dissatisfied

E. Very dissatisfied

4. What are the main problems you have encountered when using Generative AI? **(Multiple-choice question)**

A. Complex operation

B. Slow response speed

C. Unreliable generated results

D. Unfamiliarity with crafting effective prompts

E. Insufficient understanding of its functions

F. Low patient trust in AI-generated content

G. Other (Please specify): ________

5. What are your primary concerns regarding Generative AI? **(Multiple-choice question)**

A. Privacy breaches

B. Impact on autonomous clinical judgment

C. Difficulties in accountability and liability

D. Ethical conflicts and value biases

E. Risk of nursing job displacement

F. Lack of emotional support in nurse-patient interactions

G. No concerns

6. If training on Generative AI technology were available to you, which areas would interest you? **(Multiple-choice question)**

A. Basic operations and ethics of Generative AI

B. Application of Generative AI in clinical nursing practice

C. Using Generative AI to assist home-based patient care

D. Using Generative AI in nursing research

E. Application of Generative AI in nursing management systems

F. Application of Generative AI in nursing education

G. Other (Please specify): ________

Part 5: Open-Ended Questions

1. In clinical nursing work, what functions do you most wish Generative AI possessed? ________

2. What advantages do you see in the application of Generative AI in nursing work? ________

3. What shortcomings or deficiencies do you perceive in the application of Generative AI in nursing work? ________

4. What suggestions do you have regarding Generative AI? ________
