# Supplementary material for: Exploring the application of generative artificial intelligence in nursing: a cross-sectional study
Source: Front Public Health. 2026 Jan 28;14:1689418. doi: 10.3389/fpubh.2026.1689418 (PMC12893205; doi:10.3389/fpubh.2026.1689418)
Supplement: Supplementary file 2 [file Data_Sheet_2.pdf]

Appendix B

TABLE B-1 Variations in GAI Use by Demographic Characteristics (n=181)

Category	Item	Have you ever used GAI? (%)		Total	χ2	*p*
		Yes	No			
Age	<25	6(46.15)	53(31.55)	59(32.60)	3.59	0.309
	25-35	3(23.08)	82(48.81)	85(46.96)		
	36-45	3(23.08)	28(16.67)	31(17.13)		
	>45	1(7.69)	5(2.98)	6(3.31)		
Years of experience	<1 year	6(46.15)	51(30.36)	57(31.49)	3.966	0.265
	1-5 years	1(7.69)	52(30.95)	53(29.28)		
	6-10 years	1(7.69)	20(11.90)	21(11.60)		
	>10 years	5(38.46)	45(26.79)	50(27.62)		
Education attainment	Associate degree	1(7.69)	7(4.17)	8(4.42)	0.774	0.856
	Bachelor's degree	9(69.23)	127(75.60)	136(75.14)		
	Master's degree	3(23.08)	31(18.45)	34(18.78)		
	Doctoral degree	0(0.00)	3(1.79)	3(1.66)		
Type of nurse	Nurse intern	4(30.77)	25(14.88)	29(16.02)	2.331	0.312
	Staff nurse	8(61.54)	131(77.98)	139(76.80)		
	Nurse manager	1(7.69)	12(7.14)	13(7.18)		

* $p<0.05$ ** $p<0.01$ *** $p<0.001$

TABLE B-2 Factors Influencing AI Usage by General Demographic Characteristics(n=168)

Category	B (Coeff.)	SE	*z*	Wald $\chi 2$	*p*	OR	95% CI
Age	0.107	0.629	0.171	0.029	0.864	1.113	0.325 ~ 3.817
Years of experience	0.148	0.435	0.341	0.116	0.733	1.160	0.495 ~ 2.719
Education attainment	-0.181	0.632	-0.286	0.082	0.775	0.834	0.242 ~ 2.882
Type of nurse	-1.032	0.784	-1.316	1.732	0.188	0.356	0.077 ~ 1.657
Intercept	-0.823	2.102	-0.392	0.153	0.695	0.439	0.007 ~ 27.010

Note: Dependent variable = Use of GAI

TABLE B-3 Variations in GAI Use Frequency, Accuracy, and Attitudes by Demo graphic Characteristics(n=168)

Category	Item	Frequency of G AI Use	Accuracy of GAI	Attitudes toward GAI
Age	<25	2.98±1.38	2.28±0.53	2.23±0.47
	25-35	3.04±1.48	2.18±0.59	2.13±0.64
	36-45	3.86±1.35	2.14±0.71	2.00±0.61
	>45	4.00±1.00	2.00±0.71	2.00±0.71
	F	3.317	0.636	1.004
	p	0.021*	0.593	0.392
Years of ex perience	<1year	2.61±1.36	2.37±0.49	2.27±0.49
	1-5 years	3.00±1.40	2.13±0.56	2.13±0.60
	6-10 years	3.80±1.20	2.25±0.55	2.30±0.66
	>10 years	3.78±1.41	2.07±0.72	1.91±0.60
	F	7.501	2.504	3.835
	p	0.000***	0.061	0.011*
Education attainment	Associate degree	2.71±2.14	1.29±0.49	1.86±0.69
	Bachelor's degree	3.43±1.35	2.24±0.58	2.13±0.60
	Master's degree	2.32±1.30	2.23±0.50	2.16±0.52
	Doctoral degree	3.00±2.00	2.67±0.58	2.67±0.58
	F	5.521	6.977	1.365
	p	0.001**	0.000***	0.255
Type of nurse	Nurse intern	2.68±1.35	2.40±0.50	2.20±0.41
	Staff nurse	3.24±1.47	2.16±0.61	2.10±0.61
	Nurse manager	3.58±1.24	2.25±0.62	2.42±0.67
	F	2.117	1.761	1.784
	p	0.124	0.175	0.171

* $p<0.05$ ** $p<0.01$ *** $p<0.001$

TABLE B-4 Impact of General Demographic Characteristics on GAI Usage Frequency(n=168)

Category	Unstandardized Coefficients		Standardized Coefficients	*t*	*p*	Collinearity Diagnostics	
	B	**Standard Error**	***Beta***			**VIF**	***B***
Constant	3.024	0.684	-	4.419	0.000***	-	-
Age	-0.271	0.226	-0.145	-1.198	0.233	2.753	0.363
Years of experience	0.507	0.152	0.412	3.326	0.001**	2.882	0.347
Education attainment	-0.307	0.212	-0.110	-1.447	0.150	1.083	0.923
Type of nurse	0.081	0.256	0.026	0.317	0.752	1.267	0.789
F	F (4, 163) =6.322, p=0.000						
D-W	1.937						

Note: Dependent variable = Frequency of GAI use

* p<0.05 ** p<0.01 *** p<0.001
